# Enhanced Killing of Colon Cancer Cells by Mesoporous Silica Nanoparticles Loaded with Ellagic Acid

**DOI:** 10.3390/nano15201547

**Published:** 2025-10-10

**Authors:** Khaled AbouAitah, Amr Nassrallah, Ahmed A. F. Soliman, Anna Swiderska-Sroda, Tadeusz Chudoba, Julita Smalc-Koziorowska, Beom Soo Kim, Witold Łojkowski

**Affiliations:** 1 Department of Chemical Engineering, Chungbuk National University, Cheongju 28644, Republic of Korea; 2 Medicinal and Aromatic Plants Research Department, Pharmaceutical and Drug Industries Research Institute, National Research Centre (NRC), 33 El-Behouth Street, Dokki, Giza 12622, Egypt; 3 Biotechnology Program, Faculty of Basic Applied Science Institute, Egypt-Japan University of Science and Technology (E-JUST), New Borg El-Arab, Alexandria 21934, Egypt; amotagly@cu.edu.eg; 4 Biochemistry Department, Faculty of Agriculture, Cairo University, Giza 12613, Egypt; 5 Pharmacognosy Department, Pharmaceutical and Drug Industries Research Institute, National Research Centre (NRC), 33 El-Behouth Street, Dokki, Giza 12622, Egypt; ashehabeldin2007@yahoo.com; 6 Laboratory of Nanostructures and Nanomedicine, Institute of High Pressure Physics, Polish Academy of Sciences, Sokolowska 29/37, 01-142 Warsaw, Poland; a.swiderska-sroda@labnano.pl (A.S.-S.); t.chudoba@labnano.pl (T.C.); 7 Laboratory of Semiconductor Characterization, Institute of High Pressure Physics, Polish Academy of Sciences, Sokolowska 29/37, 01-142 Warsaw, Poland

**Keywords:** nanoformulation, ellagic acid, colon cancer, mesoporous silica nanoparticles, apoptosis, receptor tyrosine kinases

## Abstract

Background: Natural compounds, including ellagic acid (ELG), are promising anticancer agents with low adverse effects. In this paper, we test in vitro the effectiveness of mesoporous silica nanoparticles (MSN) as an ELG carrier against colon cancer. Methods: We produced MSNs functionalized with triptycene (TRP) and loaded with ELG, further called MSNTRPELG nanoformulation. The nanoformulation contained over 11 wt.% TRP and approximately 25 wt.% ELG in the mesoporous structure and on the surface of particles. It was assessed for anticancer effects against two colon cancer cells: HCT-116 and HT-29 for treatment with up to 200 µM. Results: Comparing to free ELG, we have shown a three times higher cancer inhibition. The lowest IC50 values were for HCT-116 (88.1 ± 0.1 µM) and HT-29 (77.6 ± 0.1 µM). When treated with free ELG, the values were 187.1 ± 0.1 µM and 300.0 ± 0.1 µM, respectively. MSNTRPELG enhanced apoptosis primarily by activating caspase-3, p53, and Bax while downregulating Bcl-2 in HCT-116 and HT-29 cells. It also inhibited receptor tyrosine kinases (HER2 and VEGFR2). Preliminary Western blot observations suggest suppression of B-RAF, C-RAF, and K-RAS oncogenes, with stronger inhibition by the nanoformulation than by free ELG. Conclusions: This work highlights the potential of MSNs to enhance the efficacy of natural prodrugs, particularly ELG, in cancer therapy.

## 1. Introduction

With extraordinary advances in cancer therapy through many cutting-edge technologies, there is a growing attention to explore the natural therapeutic agents and their therapeutic utility in clinical applications and have been studied for more than a half-century [1]. Natural agents are potentially a preferred agent against cancers as they affect signalling pathways involved in cancer initiation and progression. They have the potential to offer a safe and cost-effective treatment with reduced side effects [2]. Despite these advantages, they present also several shortcomings: physicochemical (e.g., poor solubility and stability) [3,4], pharmacokinetic limitations (e.g., poor absorption and low bioavailability) [5], and clinical application’s challenges (e.g., insufficient tumour targeting as well as low release control) [6,7]. Exploring substances derived from natural sources, such as plants, microbes, and marine sources, has garnered a lot of attention in the ongoing search for natural anticancer therapy [8]. Semisynthetic modifications of natural compounds are gaining popularity as a way to decrease toxicity and drug resistance while improving pharmacokinetics, solubility, lipophilicity, and selectivity [9].

Nanotechnology as well may permit us to overcome these barriers. The nanotechnology approach is to use nanosized particles as drug carriers to design delivery systems [7,10,11]. A branch of nanomedicine has been developed where natural therapeutic agents from plant-derived sources, e.g., flavonoids, alkaloids, and phenolic acids, are combined with a variety of nanostructures and tested for their anticancer efficiency [11,12,13]. Some nanomedicines have already been approved by the US FDA [14,15].

In previous studies, our team developed nanoformulations composed of mesoporous silica nanoparticles loaded with natural anticancer prodrugs: curcumin [16], thymoquinone [17], colchicine [18], and piperine [19]. In our recent study, we have shown that the fan inorganic–organic hybrid nanoformulation composed of zinc oxide nanoparticles functionalized with triptycene organic molecules and impregnated with ELG presented antiviral effects [20]. Of particular interest is ellagic acid (2,3,7,8-tetrahydroxy-chromeno [5,4,3-cde] chromene-5,10-dione). It is a safe plant-derived polyphenol that can be obtained from fruits, including pomegranates, green tea, grapes, strawberries, blackberries, raspberries, and walnuts [21,22], as well as from herbal materials [23]. ELG exerts biocidal, anticancer, anti-inflammatory, and antioxidant actions [22,24,25]. Numerous recent studies have shown the crucial role of ELG as a potent preventive and therapeutic agent for treating several types of cancers, including colon, prostate, breast, skin, oral, liver, and osteogenic sarcoma [26,27,28,29]. It can modulate the expression of various genes involved in cancer initiation and progression-related processes: apoptosis, proliferation, autophagy, inflammation-related genes, and oxidative-related genes [30,31]. Several reports have demonstrated its multiple mechanisms to suppress colon cancers during in vitro [32,33] and in vivo tests [34,35]. However, improved targeting and control of release are still an issue.

Most research has focused on developing nano-transporters loaded with small molecule drugs that effectively target multiple tyrosine kinase inhibitors (such as Sorafenib, Sunitinib, Afatinib, Erlotinib, and Imatinib) [36]. The main reason for using nanomedicine in this area is to obtain controlled and continuous release of such multi-inhibitors. Consequently, it reduces the drug loss and/or side effects while enabling the increased therapeutic time and effect [36]. In an earlier instance, Marslin et al. [37] developed a physically stable Imatinib Mesylate encapsulated with poly(lactide-co-glycolide) nanoparticles. The in vitro examination shows that the nanoencapsulation is more cytotoxic on MCF-7 breast cancer cells as compared to free Imatinib drug, and almost no cardiotoxic for animals was indicated. The drug-loaded nanoparticles demonstrate an effective action compared to free drug. Diniz et al. [38] demonstrated that loading nanoparticles with Foretinib causes an efficient reduction of tumor growth along with inactivated phosphorylation of targeting receptors in xenograft mice models. Many nanocarriers have been used for constructing these delivery systems (for passive and active targeting), including: liposomes, PLGA, chitosan, lipid-nanostructured, bovine serum albumin, metal–organic frameworks, MSNs, gold nanoparticles, and others [36].

The range of multifunctional nanotherapies based on inorganic materials is steadily expanding. For instance, the effective generation of reactive oxygen species through photo/chemodynamic therapy was exploited in stimulus-induced drug release [39] and hypoxia-targeted-therapy [40].

In search for ELG nanoformulations with enhanced efficacy towards cancers compared to ELG free form, Mady and Shaker [41] produced and tested ELG with poly(ε-caprolactone) as a biodegradable polymer carrier. Nanoformulation effect was evaluated in vitro on Caco-2 and HCT-116 cell lines and in vivo on New Zealand white rabbits through oral administration. The results prove a 3.6-time increase in the promotion of oral bioavailability and anticancer activity compared to the ELG free form. In another study by Ali et al. [42], one developed lactoferrin functionalized MSNs for the dual delivery of pemetrexed anticancer drug and ELG. The system shows desirable effects against breast cancer cells: overcoming the multi-drug resistance and minimizing the systemic toxicity of anticancer drugs. Kaur et al. [43] fabricated chitosan-tween 80-coated nanoformulations for ELG and have shown improvement of antitumor efficacy during in vitro and in vivo tests on breast cancer. In case of melanoma cancer, co-delivery of ELG with nanoliposomes displayed an enhanced efficiency of the PEGylated-liposomal-composed doxorubicin drug when tested in vitro and in vivo [44].

**Scheme 1:**
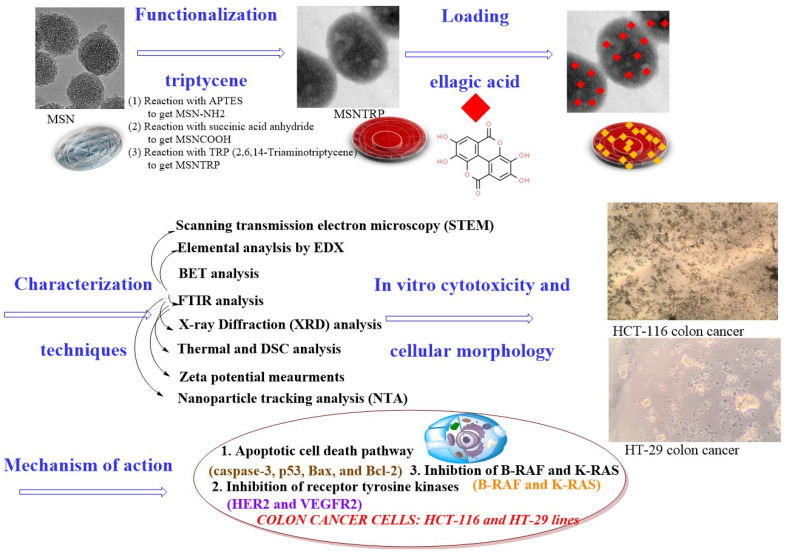
Preparation of nanoformulation, characterization techniques, in vitro studies, and mechanism of actions against colon cancer cells of HCT-116 and HT-29.

Colorectal cancer (named also colon cancer) is ranked the third top cancer and leading cause of mortality and morbidity globally [45,46]. Therefore, in the current study, we aimed to establish a nanoformulation for efficient delivery of ELG for colon cancer therapy (Scheme 1). The nanoplatforms were MSNs-functionalized with TRP. We evaluated the nanoformulation efficiency for two colon cancer cell lines (HCT-116 and HT-29). We also attempted to investigate the cancer-killing mechanisms as this may be of importance for understanding the potential cause of cancer death and for repurposing natural-based nanomedicine applications. We believe that the presented results may pave the way for the use of nanoformulations in effective anti-cancer therapies based on natural agents.

## 2. Materials and Methods

### 2.1. Mesoporous Silica Nanoparticles Synthesis, Modification, and Functionalization with Triptycene

The MSNs were synthesized in the same way as in our previous study [17] with some modifications of the procedure [47]. To prepare MSNs functionalized with TRP, we followed a multi-step synthesis as described in [20]. In the first step, MSNs were reacted with the 3-aminopropyltriethoxysilane (APTES) to obtain MSNs with NH2 molecules on the surface. For this, 1.25 g of MSNs were dispersed in 150 mL of anhydrous toluene (POCH, Gliwice, Poland), followed by slowly adding 1.5 mL of APTES (Sigma-Aldrich, St. Louis, MO, USA) under stirring with a medium speed of 250 rpm (DAIHAN Scientific, Seoul, Korea) at room temperature (RT). Next, the modified MSNs were collected through a cooling centrifuge (3–30 KS Sigma Laborzentrifugen GmbH, Osterode am Harz, Germany), then rinsed three times with distilled water (18.2 MW, Milli-Q^®^ system, Millipore, Darmstadt, Germany). Washing was done to remove unreacted APTES. Finally, the collected nanoparticles were dried via an oven at 50 °C and the final, dry product was designated as MSNNH_2_. In the second step, the MSNNH_2_ was reacted with succinic acid anhydride to produce carboxylic groups (–COOH) on the nanoparticles surface [48]. For this purpose, 1 g of MSNNH_2_ was dispersed in 50 mL of acetone (Fisher Scientific, Loughborough, UK) by sonication in a water bath for 5 min (Elma GmbH, Singen, Germany). The suspension was stirred at room temperature for 4 h at 350 rpm (DAIHAN Scientific, Seoul, Korea). Then, 40 mL (0.7 M) of succinic acid anhydride (SAA) dissolved in acetone (SAA, Acros Organics, Geel, Belgium) were added dropwise to the continuously stirred suspension. The reaction was left for 24 h at room temperature, after which the functionalized particles were centrifuged and washed with double-distilled water and methanol (Fisher Scientific, Loughborough, UK). Finally, the resulting nanoparticles were left in an oven dryer for 12 h at 60 °C to ensure the dryness and designated as MSNCOOH. The TRP-functionalized MSN were achieved in a multi-step procedure via EDC/NHs coupling chemistry. Step I). Preparation of solution A: the MSNCOOH was activated with 0.5% 1-(3-dimethylaminopropyl)-3-ethylcarbodiimide hydrochloride (EDC) and N-hydroxysuccinimide (NHS) in acetone (25 mL) (EDC and NHS, Acros Organics, Geel, Belgium); the activation was kept under stirring speed of 400 rpm for 6 h at 40 °C. Step II) Preparation of solution B: in separate screw cap bottle, 300 mg of TRP (2,6,14-Triaminotriptycene) were prepared from triptycene due to procedure described in detail in our study [20] and was activated also in acetone containing EDC/NHS (0.5%, 50 mL) using condition done for solution A. Step III). Reaction between MSNCOOH and TRP was achieved by addition dropwise of 25 mL of solution B into solution A, then the mixture was stirred (250 rpm) at RT for 24 h. Finally, the nanoparticles were collected by centrifugation, washed with double-distilled water and acetone (aiming to remove unreacted TRP molecules) and dried in an oven at 60 °C. The obtained material was designated MSNTRP.

### 2.2. Nanoformulation Preparation

We prepared the nanoformulations with a drug to carrier ratio of 1:1.5. For that, the 100 mg of ELG (Sigma-Aldrich, St. Louis, MO, USA) were dissolved in 10 mL of acetone under stirring condition. Then, 150 mg of MSNTRP were added and stirred at 170 rpm at RT for 24 h. Thereafter, the acetone was evaporated at 50 °C in a Rotavapor (Büchi, Flawil, Switzerland) until dryness, followed by heating at an oven for 12 h at 60 °C. Dried nanoformulation was named MSNTRPELG.

### 2.3. Nanostructure Characterization Techniques

Scanning transmission electron microscopy (STEM) and TEM (transmission electron microscopy) studies were carried out using the FEI TECNAI G2 F20 S-TWIN (Thermo Fisher Scientific, Waltham, MA, USA) microscope. By the analysis, we obtained TEM and STEM images of materials. To determine the elemental composition of the obtained materials, we used FE–SEM (Ultra Plus, Zeiss, Jena, Germany) coupled with energy-dispersive X-ray spectroscopy (EDX). The particles were suspended in deionized water, sputtered on a silicon wafer, and dried. To measure the specific surface area (SSA), we used the Brunauer-Emmett-Teller (BET) method under ISO 9277:2010 (Gemini 2360, Micromeritics, Norcross, GA, USA). Before analysis, the nanopowders without ELG were degassed at 150 °C in flowing helium for 12 h. Degassing of MSNTRPELG was performed at 50 °C under constant helium flow for 24 h using a FlowPrep 060 desorption station (Micromeritics). To track the changes of the surface functional groups on MSNs after subsequent treatments, we utilized the Fourier transform infrared (FTIR) spectroscopy coupled with attenuated total reflectance (ATR) using the Bruker Tensor 27 IR instrument and Bruker Platinum ATR-Einheit A 255 (Bruker Corporation, Billerica, MA, USA). X-ray Diffraction (XRD) analysis was conducted using a powder XRD (X’PertPRO System, PANalytical, Marietta, GA, USA) with the following condition: a CuKα radiation with a 10–100° 2θ range. Simultaneous Thermal Analysis was carried out using a 449 F1 Jupiter^®^ (NETZSCH-Feinmahltechnik GmbH, Selb, Germany) equipment. Samples with a mass of 10 ± 1 mg were heated gradually from RT up to approximately 800 °C with a heating rate of 10 °C/min. Experiments were conducted in an artificial air and helium mixture flowing through the furnace chamber. Before the measurement, the furnace chamber was flushed with the same gas mixture for 10 min. The surface charges of the materials were measured by determining the zeta potential for the particles suspended in deionized water adjusted to neutral pH. The analysis was performed with a Laser Doppler Electrophoresis (LDE) analyser at λ = 633 nm (Zetasizer Nano-ZS ZEN 3600, Malvern Instruments Ltd., Malvern, UK). Particle size distribution of the obtained nanopowders suspended in deionized water was done using an NTA analysis by the NS500 NanoSight instrument (λ = 405 nm, Malvern Panalytical Ltd., Malvern, UK).

### 2.4. Cell Cultures, In Vitro Cytotoxicity, and Anticancer Mechanism Evaluation and Gene Expression Profiling

#### 2.4.1. Cell Cultures

The HCT-116 human colorectal carcinoma cell line and HT-29 human colorectal adenocarcinoma cell line (Karolinska Institute, Stockholm, Sweden) were maintained in RPMI 1640 supplemented with 10% heat-inactivated foetal bovine serum plus 1% antibiotic–antimycotic mixture (10,000 µg/mL of streptomycin sulphate, 25 µg/mL of amphotericin B, 10,000 U/mL of potassium penicillin, and 1% L of glutamine; Biowest, Riverside, MO, USA). Employed as a BJ-1, the human skin fibroblast cell line derived from foreskin was kindly provided by Professor Stig Linder, oncology and pathology department, Karolinska Institute, Stockholm, Sweeden that was formerly obtained from the American Type Culture Collection (ATCC). The cell was maintained in DMEM (consisting of 2 mM L-glutamine, Earle’s salts medium) (Biowest, Riverside, MO, USA). Our experiments were performed in a sterile laminar air flow cabinet of biosafety class II. All incubations took place at 37 °C under 5% CO_2_ in a 95% humidified atmosphere.

#### 2.4.2. In Vitro Studies

To perform the in vitro studies, we followed the methods reported in our previous study [49] using an MTT assay. The cells were seeded in 96-well microtiter plastic plates to a density of 104 cells per well and further incubated for 24 h. After the medium was aspirated, we added fresh medium containing MSNs up to 1000 µg/mL, MSNTRPELG nanoformulation (with an equivalent concentration to free ELG, which was up to 200 µM), and, for comparison, free ELG was used at the same concentration. The equivalent concentration of MSNTRPELG was prepared based on ELG content calculated from TGA weight loss data. After treatment of cells with MSN or MSNTRPELG, they were incubated for 72 h with DMEM alone for untreated control cells. Afterward, 40 µL of MTT salt (Bio Basic Canada Inc., Toronto, ON, Canada) at 2.5 µg/mL per well was added, followed by a 4 h incubation. The addition of 200 µL of 10% sodium dodecyl sulphate (SDS) and overnight incubation at 37 °C was used to stop the reaction and dissolve formazan crystals. Formazan product was measured on a microplate reader at 595 nm to 690 nm (reference wavelength) as background (model 3350, Bio-Rad, Hercules, CA, USA). The calculation for cytotoxicity was [(reading of sample/reading of negative control) − 1] × 100. The IC50 (concentration yielding 50% inhibition of cell viability) was calculated by applying various concentrations of treatments and the probit analysis method with a *t*-test (SPSS version 11.0, Chicago, IL, USA).

#### 2.4.3. Anticancer Mechanism Evaluation

Determination of cellular levels of key apoptosis marker proteins was conducted 24 h post-treatment with the IC50 of tested samples. Briefly, cells (HCT-116, HT-29, and BJ-1) were seeded at a concentration of 1.2–1.8 × 10^3^ cells per well in six-well plates. After 24 h treatment, the cells were trypsinised and centrifuged at 10,000 rpm for 20 min at 4 °C. After that, the cell pellets were ready for use and stored for further experiments.

#### 2.4.4. Gene Expression Profiling by RT-qPCR

Gene expression of key pro- and anti-apoptotic, in addition to tyrosine inhibitors HER2 and VEGFR-2 marker genes, was conducted for HT-29, HCT-116, and BJ-1 Fibroblast cell lines treated with the IC50 obtained from the dose-responsive concentrations. After 48 h of incubation, total RNA was extracted using of Total RNA Purification Kit (Norgen Biotek Corp., Thorold, ON, Canada) according to the manufacturer’s instructions. Synthesis of cDNA from RNA was performed using the QuantiTect Reverse Transcriptase Kit (Qiagen, Hilden, Germany). The quantitative PCR for specific targets was performed with multi-well plate Applied Biosystem 7500 (GmbH, Düsseldorf, Germany) with QuantiTect SYBR-Green PCR Kit (Qiagen, Hilden, Germany) as previously described [50]. The primer sequences used in this study are listed in Table 1. The reaction mixture of a final volume (10 µL) containing cDNA sample (2 µL), 2x SYRB-Green PCR Master Mix (4 µL), each forward and reverse primer (20 µM stock, 1.25 µL), and RNase-free water (1.5 µL). Each sample was represented by two biological replicas and three technical replicas, with the inclusion of a non-template control (NTC). Raw data were analyzed using the Applied Biosystem 7500 (GmbH, Düsseldorf, Germany) cycler software 2.1 to calculate the threshold cycle (Ct) using the second derivative maximum. The fold-change value for each gene was determined after normalization to the expression levels of GABDH as an HK gene, which was calculated using the equation 2^−ΔΔCt^.

### 2.5. Extraction of Protein and Western Blot Analysis

Immunoblotting analyses were conducted as previously described [51]. After 72 h of incubation, cells were collected and total proteins were extracted using a buffer composed of 150 mM of NaCl, 50 mM of Tris-HCl, pH 7.5, 10 mM of MgCl_2_, 1 mM of PMSF, 0.1% NP-40, and 1x complete protease inhibitor (Roche). The cell lysate was centrifuged twice at 14,000× *g* for 10 min at 4 °C, and the supernatants were transferred into a new tube. Protein concentration was determined by the Bradford protein assay [52], and then 10 µg of protein extract was loaded into 10% SDS-PAGE gels. Gels were transferred to a PVDF membrane using a semidry transfer blot system. Membranes were then blocked and subjected to immunodetection with the anti-p53 monoclonal antibody (PAb 240; ab26; Abcam, Cambridge, UK), anti-Bcl-2 monoclonal antibody (ab32124; Abcam, UK), anti-Bax monoclonal antibody (E63, ab32503; Abcam, UK), Anti-Caspase-3 polyclonal antibody (ab13847; Abcam, UK), Anti-B-Raf (ab308176; Abcam, UK), Anti-c-Raf (ab137435; Abcam, UK), Anti-K-Ras (ab275885; Abcam, Cambridge, UK), and anti-β Actin monoclonal antibody (SP124; ab115777; Abcam, UK) antibodies. The secondary antibodies used were: Anti-Mouse IgG (Amersham Biosciences, Buckinghamshire, UK) and Anti-Rabbit (GE Healthcare, Milwaukee, WI, USA).

Densitometric analysis: Western Blots were further quantified using ImageJ (rolling-ball background subtraction). For each lane, target protein signals were normalized to β-actin and expressed relative to the untreated control (set to 1.0).

### 2.6. Statistical Analysis

The cytotoxicity, anticancer, and anticancer mechanisms data are given as mean value ± standard deviation (SD). Significant differences in cytotoxicity and anticancer effects were evaluated by one-way analysis of variance (One-sample Wilcoxon test and Paired *t*-test; GraphPad PRISM, v 8.0.1, San Diego, CA, USA). All molecular evaluations were statistically analysed using one-way ANOVA through the least-significant differences according to Assaad et al. [53]. For Western blot analyses, single experiments per condition were available. Densitometric values are presented descriptively without calculation of variability or inferential statistics. Densitometric quantification of the available blots was performed, normalized to β-actin and control, and presented the results as fold-change values. The data are presented without hypothesis-testing statistics.

## 3. Results and Discussion

### 3.1. Microscopical Observations

**Figure 1:**
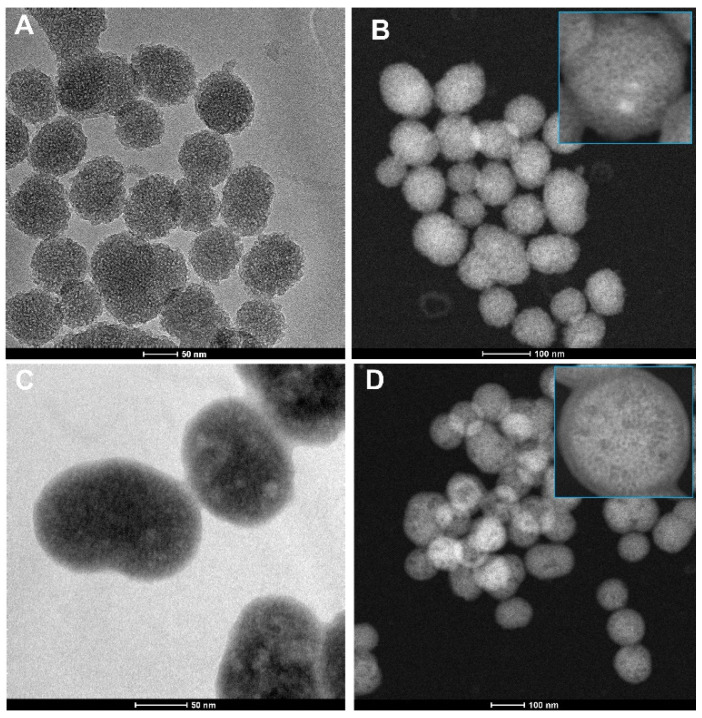
Electron microscope images before and after functionalization of MSNs. (**A**) TEM image of MSNs, (**B**) STEM image of MSNs, (**C**) TEM image of MSNTRP, and (**D**) STEM image of MSNTRP.

TEM images presented in Figure 1A,C show a substantial difference in the morphology of the MSNTRP compared to MSN. STEM observations revealed presence of coating layer(s) on MSNs after TRP treatment, as seen in Figure 1B,D. In summary, microscope observations showed a strong attachment of TRP molecules to the MSN surface and confirmed that the functionalization process was successful. This result is consistent with our data obtained for zinc oxide nanoparticles functionalized with TRP, employed as a hybrid nanosystem for delivery of ELG [20].

### 3.2. Nanoparticle Tracking Analysis (NTA)

**Figure 2:**
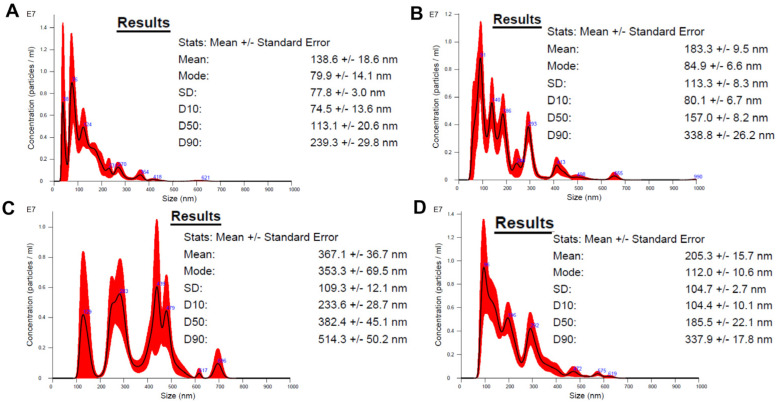
Nanoparticle tracking analysis of (**A**) MSN, (**B**) MSNCOOH, (**C**) MSNTRP, and (**D**) MSNTRPELG.

Figure 2 shows the following sequence of particle size as measured using the NTA technique: MSN (138.6 ± 18.6 nm), MSNCOOH (183.3 ± 9.5 nm), and MSNTRP (367.1 ± 36.7 nm). After loading ELG into nanoparticles (MSNTRPELG), a decrease in the measured using this method size of the nanoformulation was recorded (205.3 ± 15.7 nm).

Interestingly, after ELG loading, the average particle size decreased from 367.1 ± 36.7 nm for MSNTRP to 205.3 ± 15.7 nm for MSNTRPELG, as measured by NTA (Figure 2). Although this effect may appear counterintuitive, similar findings have been reported for other natural compounds, such as quercetin-loaded MSNs [54], as well as for spray-dried PulmoSphere™ formulations with high drug loading [55]. A plausible explanation is provided by the change in surface charge: the zeta potential of MSNTRPELG (−55 mV) was markedly more negative than that of MSNTRP (−29 mV). A higher negative surface charge increases electrostatic repulsion, reduces nanoparticle agglomeration, and enhances colloidal dispersion. Because NTA measures hydrodynamic size based on diffusion speed in suspension, reduced aggregation is recorded as a smaller apparent particle size [56]. The synthesis method applied here, therefore, yielded MSNTRPELG particles that remain well dispersed and stable in aqueous suspension, which is advantageous for biomedical applications [57].

### 3.3. Elemental Analysis

**Figure 3:**
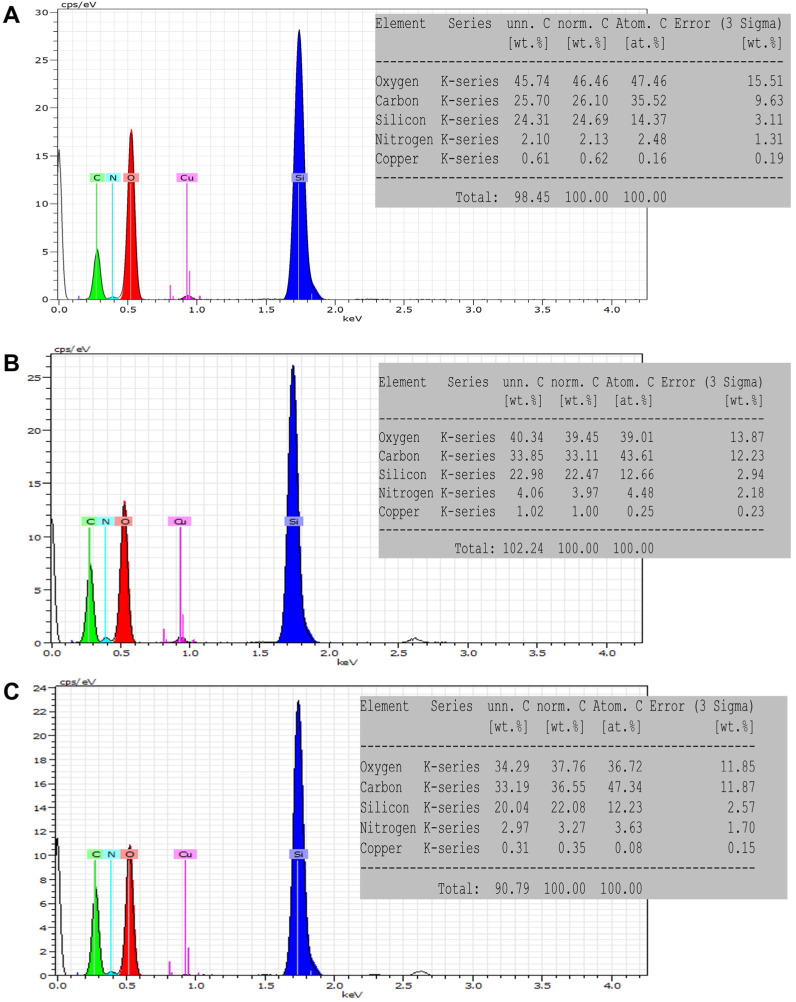
Chemical analysis utilizing EDX to measure the elemental content in functionalised nanoparticles. (**A**) MSNCOOH, (**B**) MSNTRP, and (**C**) MSNTRPELG.

We used FE-SEM-coupled with EDX to determine the chemical composition of the materials after surface modification and drug loading. Figure 3 shows that the average elemental content of the main elements (O, Si, N, and C), as detected in two places of each sample, varied. The EDX analysis indicated that C and N contents increased after functionalization with TRP (33.1 wt.% and 3.9 wt.%, respectively) and loading with ELG (36.5 wt.% and 3.2 wt.%, respectively) compared to MSNCOOH (26.1 wt.% and 2.1 wt.%, respectively). A registered increase of N and C amounts in MSNTRP and MSNTRPELG was expected due to the organic content of TRP and ELG. These results highlight the achievement of ELG loading of the MSNs.

### 3.4. FTIR-ATR Analysis

**Figure 4:**
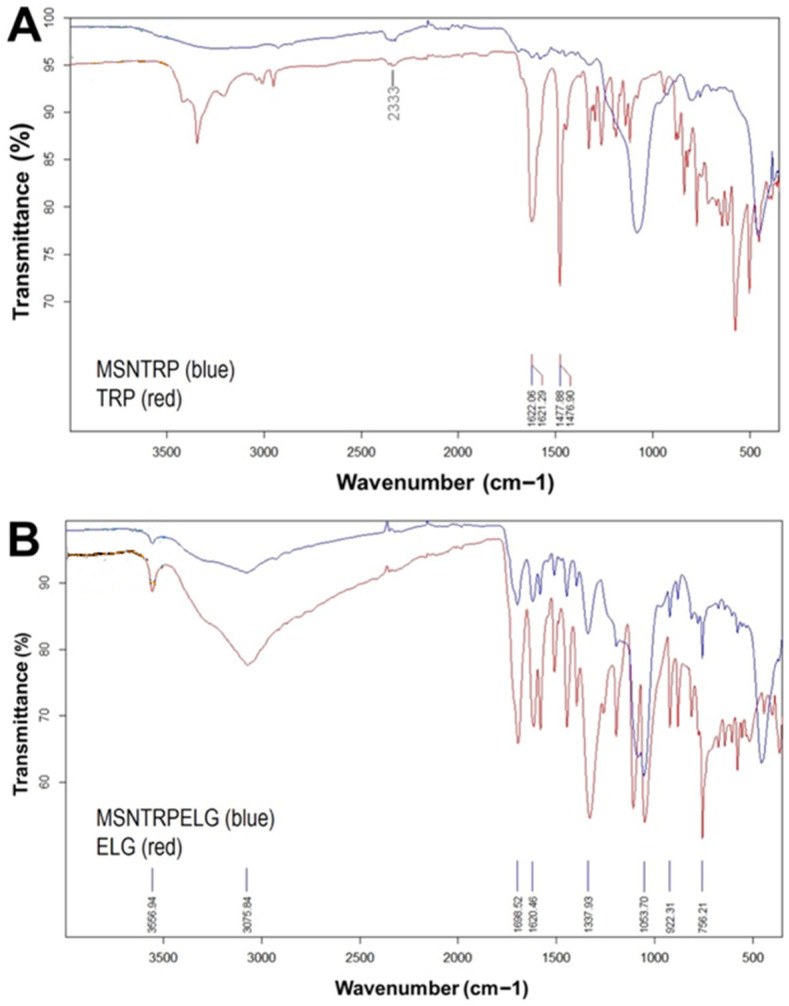
ATR-FTIR spectra of nanoparticles, nanoformulation, and free ELG. (**A**) FTIR spectra of TRP and functionalized nanostructures, and (**B**) FTIR spectra of nanoformulation and free ELG.

The results of the FTIR-ATR analysis are presented in Figure 4. Three weak signals corresponding to the bands of pure TRP at 1478 cm^−1^, 1622 cm^−1^, and 2333 cm^−1^ are visible in the FTIR spectrum of the MSNTRP sample (Figure 4A). Considering TG results, showing that the TRP content in MSNTRP is as high as 12 wt.% (see Table 2), it can be supposed that TRP molecules were not only attached to the surface of nanoparticles but also distributed into the mesoporous structure of MSNCOOH, which agrees with our previous investigations [20]. In turn, results obtained for the MSNTRPELG sample clearly showed the presence of ELG molecules on the surface of nanoparticles (Figure 4B)**.** A set of peaks coming from pure ELG (e.g., at 756, 922, 1054, 1338, 1620, 1699, 3076, 3557 cm^−1^) is present in FTIR data of MSNTRPELG (Figure 4B). Signals at 1620 cm^−1^ and 1699 cm^−1^ reflect C=C stretching vibrations and carboxylic band (C=O), respectively. While bands at 3076 cm^−1^ and 3557 cm^−1^ may indicate stretching of the hydrogen in an aromatic ring and O–H stretching vibrations in ELG, respectively [58,59]. Thermal analysis showed that the amount of ELG in MSNTRPELG reached 25 wt.% (see Table 2); therefore, FTIR data markedly confirm that the loading of ELG into/on the hybrid nanostructure succeeded. Present results are consistent with those previously obtained by us for ZnO nanoparticles modified with TRP and loaded with ELG, as well as for protocatechuic acid-loaded functionalized ZnO nanoparticles [20,60]. The loading of ELG to functionalized nanoparticles can take place through intermolecular non-covalent interactions, mainly by various hydrogen bonding and π-π stacking as described in our recent study [20].

### 3.5. XRD Analysis

**Figure 5:**
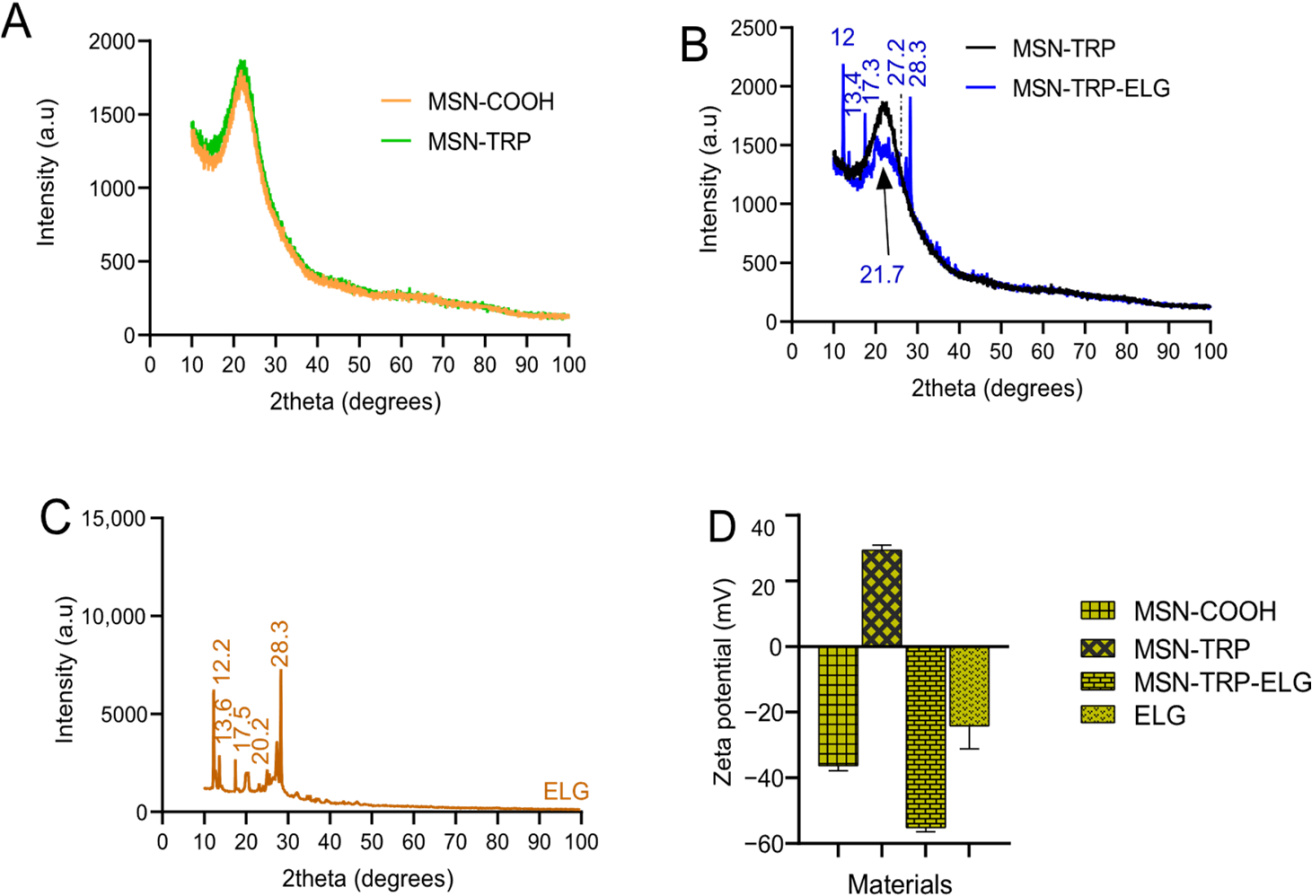
XRD patterns and zeta potential measurements of nanoparticles, nanoformulation, and free ELG. (**A**) XRD patterns of nanostructures before and after functionalization, (**B**) XRD pattern of nanoformulation compared to functionalized nanoparticles, (**C**) XRD pattern of free ELG, and (**D**) Zeta potential of all materials.

Figure 5A presents the XRD patterns for MSNCOOH and MSNTRP, revealing negligible changes in the pattern after TRP surface modification. Concerning ELG loading (Figure 5B), there were sharp reflection peaks (2θ = 12, 13.4, 17.3, 27.2, and 28.3°) found in nanoformulation, which correspond to ELG, as shown in the original pattern of pure ELG in Figure 5C. These peaks show the possibility of the presence of some ELG on the surface, which is in line with the data of FTIR-ATR.

### 3.6. Zeta Potential Analysis

The results of zeta potential analysis are presented in Figure 5D. Varied responses were observed for the different materials suspended in deionized water at pH 7.4. Among them, only MSNTRP carried a positive surface charge (+29.4 ± 1.5 mV), which is in line with our previous data for ZnO NPs modified with TRP [20]. In contrast, MSNCOOH, MSNTRPELG, and ELG exhibited negative surface charges, with zeta potential values of −36.3 ± 1.5 mV, −55.2 ± 1.3 mV, and −24.2 ± 6.9 mV, respectively. The negative charge of MSNCOOH was expected due to succinic acid modification.

Importantly, ELG loading shifted the surface potential further toward negative values, consistent with the presence of ELG on the nanoparticle surface. This observation is in agreement with the FTIR-ATR and XRD results, and similar behaviour was reported previously for ELG loading into ZnO NPs modified with TRP [20]. Surface charge is a key determinant of nanoparticle dispersion, stability, cytotoxicity, and cellular uptake [61,62], underlining the significance of these findings.

A plausible explanation is that encapsulation of ELG within the functionalized MSN carrier alters the distribution of surface functional groups. Consequently, the MSNTRPELG nanoformulation exhibited a more negative zeta potential (−55 mV) compared to free ELG (−24 mV). This suggests that the contribution of the carrier surface dominates over the hydroxyl groups of ELG, with the enhanced negative charge likely arising from exposed carboxyl and silanol groups on the MSN surface, together with improved colloidal dispersion.

### 3.7. Simultaneous Thermal Analysis (STA) and Specific Surface Area

**Figure 6:**
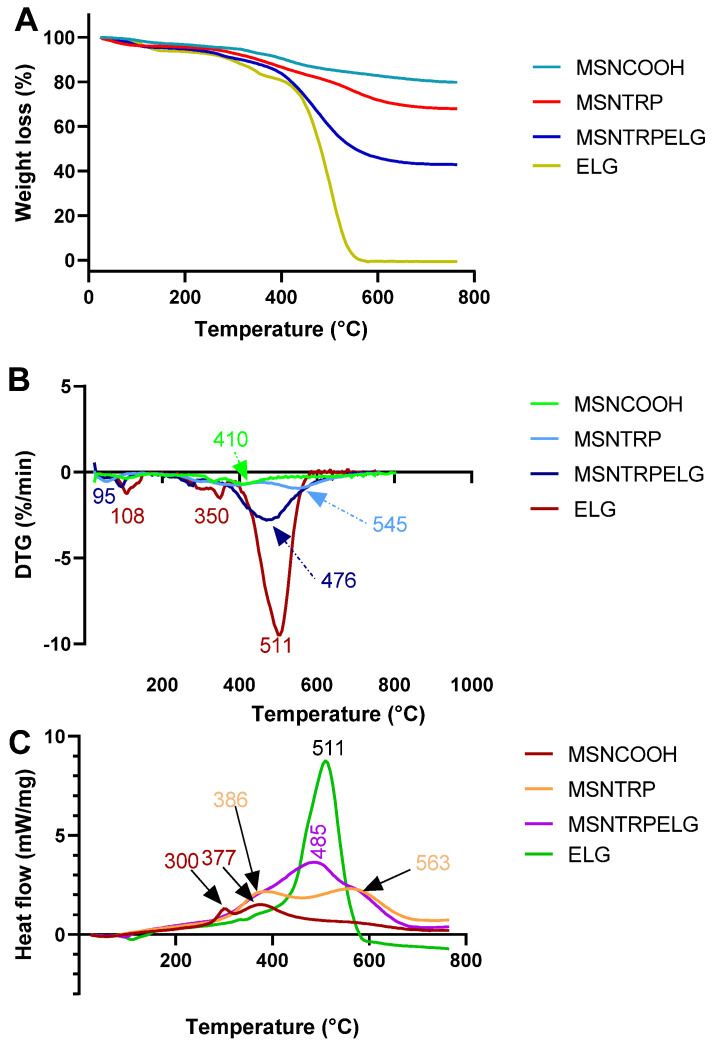
Thermal properties of nanoparticles, nanoformulation, and free ellagic acid. (**A**) TG curves, (**B**) DTG curves, and (**C**) DSC curves.

STA was performed to observe mass change (TG curve) and, at the same time, thermally induced processes (DSC curve) occurring during heat treatment of the examined materials. Results are presented in Figure 6. To follow mass change kinetics, the derivative of TG data (DTG curve) was established and presented in Figure 6B. A correlation between signals in the DSC curve and peaks in the DTG curve was found previously in our research performed with other nano-delivery systems [20,49]. Based on TG results, the percentage of TRP and ELG in specific samples, as well as loading efficiency, was calculated (see Table 2). As can be seen in Figure 6A, mass losses were recorded for all tested samples. However, the percentage of weight change depended on the material. For pure ELG, mass loss reached 100% (Figure 6A). According to DTG data, the mass loss was particularly intense in two temperature ranges: 70–160 °C and 380–600 °C (Figure 6B). This result correlates very well with the thermal effects on the DSC curve (Figure 6C). At the beginning of the experiment, an endothermic peak is observed with an extremum value near 110 °C. At higher temperatures, a strong exothermic signal with an extremum at about 510 °C is detected (Figure 6C). We assume that the endothermic one is related to the moisture release, while the exothermic one indicates the decomposition and oxidation of ELG. In the case of preparations, the percentage of mass loss increased with subsequent steps of preparation and reached 20.4 wt.% for MSNCOOH, 32.1 wt.% for MSNTRP, and 57.1 wt.% for MNSTRPELG (Table 2). Relatively large mass loss of MNSCOOH, namely 20.4 wt.%, was probably due to the significant content of succinic acid in the sample. The weight of MSNTRP decreased by 32.1 wt.%. The amount of TRP in surface-modified particles reached more than 11 wt.%. Similarly, thermal characteristics for both MSNCOOH and MSNTRP samples were found (see Figure 6B,C). At the temperature range RT-100 °C, a broad peak connected with water evaporation is observed in the DTG and DSC graphs. At higher temperatures, two overlapping exothermic effects were recorded in the DSC curve and corresponding peaks in the DTG curve. However, in the case of MSNTRP, exothermic peaks are importantly wider and shifted towards higher temperatures. For MSNCOOH, extreme peaks are observed at 300 and 377 °C, while for MSNTRP, at 386 °C and 563 °C (Figure 6C). Such a change results from the thermally induced decomposition of organic TRP. This indicates good interaction between MSNCOOH and TRP, enabling the formation of inorganic–organic hybrid nanoparticles. MSNTRPELG sample exhibited a mass loss of 57.1 wt.%. Hence, the amount of ELG in nanoformulation is as high as 25% by weight. Kinetics of mass change and appropriate endothermic and exothermic signals in the DSC curve are like those obtained for pure ELG (Figure 6B,C). The main difference is that the intensive exothermic peak recorded for MSNTRPELG is much wider and shifted to a lower temperature compared to that registered for pure ELG (Figure 6C). For ELG, the extreme peak value is observed at 511 °C, while for MSNTRPELG, it is observed at 485 °C (Figure 6C). Peak’s broadening and shift result from the overlapping processes occurring in all MSNTRPELG components, described earlier. It is seen that the proposed hybrid nanostructure is thermally stable and contains proper amount of TRP and ELG mainly inside the nano-delivery system.

The specific surface area of the materials after each step of fabrication was measured. It is seen that the specific surface area decreased after TRP functionalization and ELG loading (Table 2). This observation is expected and indicates successful preparation to obtain the final nanoformulation.

### 3.8. Loading Efficiency

The total ELG loading (TDL) in the MSNTRPELG nanoformulations, calculated from TG data, was 25 wt.%. (Table 2). Also, the calculated EE was 62.5 wt.%. The obtained TDL and EE values are relatively high and sufficient to produce a nano-delivery system. Such properties are within the range of various nanoformulations made for ELG under several strategies, as discussed previously [63].

### 3.9. Cytotoxicity and Anticancer Effects

**Figure 7:**
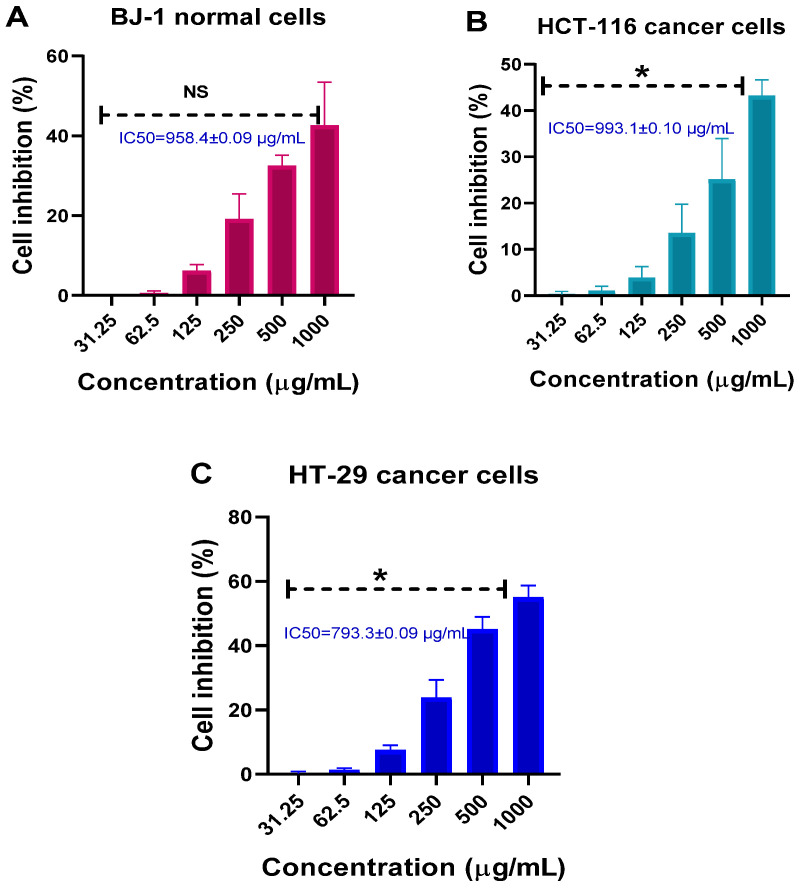
Cytotoxicity effects of MSNs on BJ-1, HCT-116, and HT-29 cell lines exposed to different concentrations and incubated for 72 h. (**A**) BJ-1 normal cells, (**B**) HCT-116 colon cancer cells, and (**C**) HT-29 colon cancer cells. Data are provided as mean ± SD. Significant diff. among means (*p* < 0.05); for BJ-1, no significant differences (*p* value: 0.062, *p* value summary: ns), as well as for HCT-116 cells (*p* value: 0.031, *p* value summary: *) and for HT-29 cells (*p* value: 0.031, *p* value summary: *) Cell viability (% of control).

Initially, we evaluated the cytocompatibility of MSNs on normal and colon cancer cell lines up to a high concentration (1000 µg/mL). As no specific normal colon cancer cells were available to us to compare with colon cancer cell lines of HCT-116 and HT-29, we checked the cytocompatibility using BJ-1. The reason is related to the practice of use of a co-culture of colon fibroblasts with skin fibroblasts like BJ-1 cells [64]. Figure 7 shows that, in cancer cells, the inhibition increased significantly (<0.05) with the increment in concentrations (Figure 7A–C). The higher inhibition percentages were noticed when cells were treated at 1000 µg/mL, indicating cell inhibition was concentration dependent. At this high concentration, the inhibition rate was about 42.6% ± 10.8, 43.2% ± 3.4, and 55% ± 3.7 for BJ-1, HCT-116, and HT-29, respectively. There were no significant differences between all normal and cancer cells subjected to MSNs at various concentrations. IC50 calculated values were found to be 958.4 ± 0.01 µg/mL, 993.1 ± 0.1 µg/mL, and 793.3 ± 0.1 µg/mL for BJ-1, HCT-116, and HT-29, respectively.

**Figure 8:**
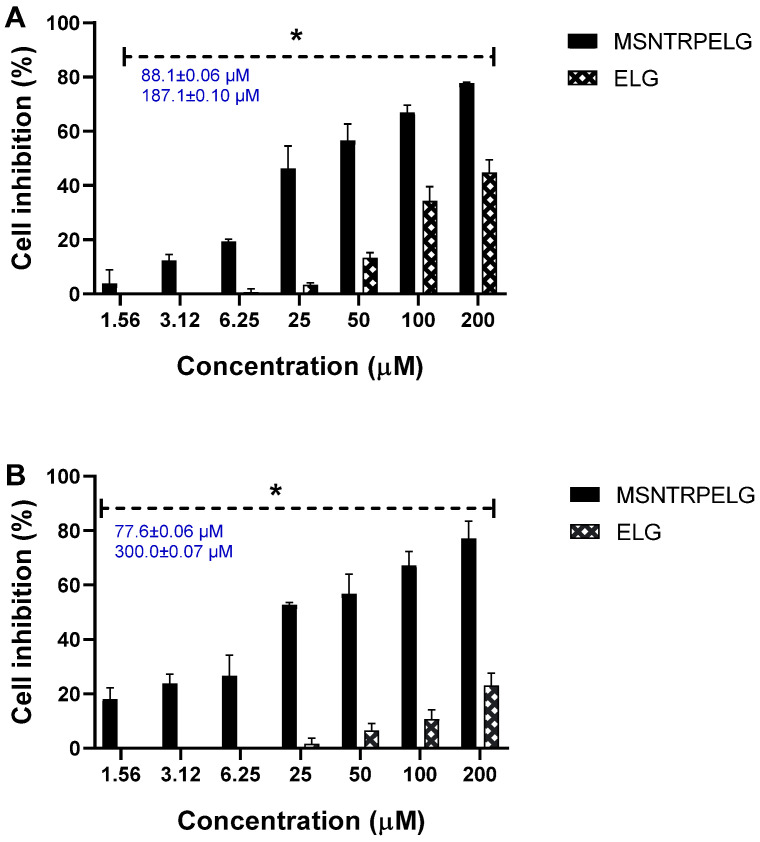
Anticancer effects of nanoformulation and free ELG on HCT-116 and HT-29 cell lines exposed to different concentrations and incubated for 72 h. (**A**) HCT-116 cells and (**B**) HT-29 cells. Data are provided as mean ± SD. * Significant differences at *p* < 0.05; Cell viability (% of control).

The results of the anticancer evaluations, for which the nanoformulations and free ELG were tested, are shown in (Figure 8). Obviously, as concentration was increased, MSNTRPELG significantly better inhibited HCT-16 and HT-29 colon cancer lines compared to free ELG. In HCT-116, at a high concentration of 200 µM, the inhibition percentage was 77.8% ± 0.3 with nanoformulation and 44.7% ± 4.8 with free ELG (Figure 8A). Also, in HT-29 cells treated with 200 µM, it was inhibited to 77.1% ± 6.5 by nanoformulation and 23.0% ± 4.6 by free ELG (Figure 8B). IC50 data showed the lowest values for nanoformulations in contrast to free ELG, as follows: nanoformulation (88.1 ± 0.01 µM for HCT-116 and 77.6 ± 0.1 µM for HT-29) and ELG (187.1 ± 0.1 µM for HCT-116 and 300.0 ± 0.1 µM for HT-29). Thus, the anticancer effects of nanoformulation were up to about three times stronger than for ELG alone.

The enhanced anticancer effect observed for the MSNTRPELG nanoformulation compared to free ELG can be explained by several complementary mechanisms [6]. Many small molecules, including ELG, suffer from low aqueous solubility and poor stability. MSNs are readily dispersible in water, and since they carry ELG within their pores, they enable its transport in aqueous environments where free ELG alone is poorly dispersible. Further, MSN encapsulation may protect ELG from degradation, leading to higher bioavailability at the cellular level. Furthermore, MSNs may facilitate cellular uptake through endocytosis and provide a sustained release of the drug, as shown for example in ref. [16]. These properties prolong the exposure of tumour cells to ELG and enhance its inhibitory action on the RAF/RAS-signalling pathway. Free ELG is rapidly metabolized/cleared, limiting its intracellular exposure time. MSNs provide sustained release, maintaining therapeutic intracellular concentrations, allowing more effective inhibition of oncogenic pathways. Further, free ELG must cross cell membranes by passive diffusion, often inefficient. Nano-sized MSNs are internalized by cancer cells via endocytosis, resulting in higher intracellular drug concentrations compared to free drug in the medium. Surface functionalization of MSNs may improve targeting and reduce drug efflux [65,66].

These results are in line with other studies of ELG-based nanoformulations. When tested in vitro, Mady and Shaker found that encapsulating ELG in biodegradable polymeric nanoparticles improved oral bioavailability, which led to effective anticancer activity, cellular uptake, and efficient localization in the nuclear region of Caco-2 cells [41]. Another study found that ELG-encapsulated nano-sized metallacages inhibit cancer cells (A549 human lung cell line) via modulating mRNA induction and protein expression levels when compared to their free form [67]. In addition, the pharmacokinetic characteristics for ellagic acid-hollow plasticized zein nanoparticles showed a 3.6- and 2.1-fold improvement in bioavailability when compared to ELG solution and ELG solid nanoparticles, indicating that the creation of ELG nanoformulation can be improved [68].

### 3.10. Cellular Morphology Observation

**Figure 9:**
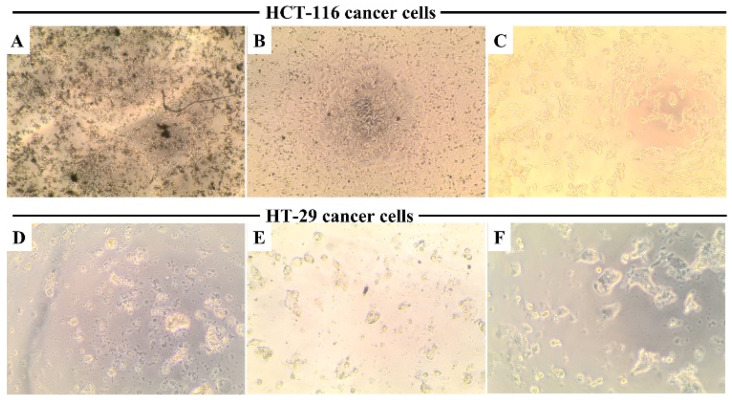
Cell morphology changes after the treatment with nanoformulation and free ELG compared to control. (**A**) HCT-116 treated with nanoformulation, (**B**) HCT-116 treated with ELG, (**C**) HCT-116 without treatment, (**D**) HT-29 treated with nanoformulation, (**E**) HT-29 treated with ELG, and (**F**) HT-29 without treatment. Incubation was 72 h.

Figure 9 shows that cellular morphology in HCT-16 and HT-29 cells is directly affected by MSNTRPELG (Figure 9A,D) and free ELG (Figure 9B,E) when compared to untreated control cells (Figure 9C,F). Untreated cancer cells were homogeneously distributed on a cultured field, exhibiting a uniform polygonal shape, and no significant morphological change. Following the incubation with, especially, MSNTRPELG and free ELG, the shapes of the cells were transformed from polygonal to circular, resulting in cell shrinkage. This observation agrees with previous data for breast cancer cells treated with nanoparticles composed of tannin [69]. Notably, these morphological alterations were observed at 200 µM. It is seen that the treatment of cells with nanoformulations caused stronger shrinkage when compared to the treatment with free ELG.

### 3.11. Evaluation of the Mechanism of Action

#### Apoptotic Cell Death Pathway

**Figure 10:**
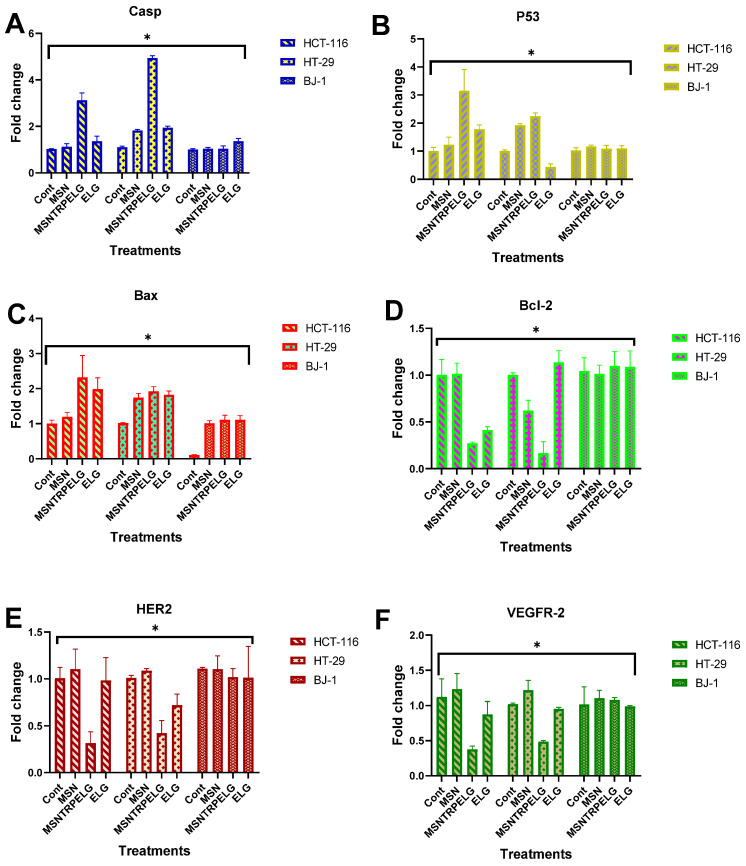
Apoptotic cell death and receptor tyrosine inhibition. (**A**) Caspase-3 expression, (**B**) p53 expression, (**C**) Bax expression, (**D**) Bcl-2 expression, (**E**) HER2 expression, and (**F**) VEGFR-2 expression. Data are provided as mean ± SD. ANOVA one way by LSD (least significant differences). The cells were incubated for 72 h. * Significant differences at *p* < 0.05.

Apoptotic cell death occurs through multiple pathways, including caspase-3, p53, Bax, and Bcl-2, which are central to the apoptosis process. In this study, we assessed gene expression in HCT-116, HT-29, and BJ-1 cells before and after treatment. As shown in Figure 10A, MSNTRPELG demonstrated a significant upregulation of pro-apoptotic markers, including caspase-3 expression, with a 3.1 ± 0.3-fold change in HCT-116 cells and a 4.9 ± 0.1-fold change in HT-29 cells compared to untreated controls. In contrast, free ELG exhibited a higher level of caspase-3 expression compared to MSN and MSNTRPELG. Figure 10B illustrates a significant increase in p53 expression in HCT-116 and HT-29 cells when treated with MSNTRPELG, reaching 3.1 ± 0.7-fold change and 2.2 ± 0.1-fold change, respectively. However, no significant p53 gene expression was detected in BJ-1 cells after treatment. As shown in Figure 10C, Bax expression was significantly enhanced in both HCT-116 and HT-29 cells exposed to MSNTRPELG, with fold changes of 2.3 ± 0.6 and 1.9 ± 0.1, respectively. No significant differences were observed in BJ-1 cells treated with MSN, ELG, or the nanoformulation. Additionally, the expression of the anti-apoptotic marker Bcl-2 was significantly downregulated in HCT-116 and HT-29 cells treated with MSNTRPELG, showing fold changes of 0.27 ± 0.01 and 0.17 ± 0.12, respectively, compared to the control (Figure 10D). As expected, no significant differences were observed in BJ-1 cells across all treatments. These results suggest that MSNTRPELG has the potential to enhance apoptosis induction in cancer cells, marked by the elevated expression of caspase-3, p53, and Bax, as well as a significant decrease in Bcl-2.

Similarly, in an earlier study by Chang et al. [70], fabricated PLGA-curcumin nanoparticles triggered the intrinsic apoptotic pathway by increasing the activity and expression levels of caspase-3, caspase-9, and Bax while downregulating Bcl-2 in CAL27-cisplatin-resistant human oral cancer cells. A previous study also confirmed that a PLGA-quercetin nanoformulation inhibited cervical and breast cancer progression [71]. Furthermore, compared to free berberine, BBM-NPs showed stronger inhibitory effects on xenograft tumours derived from PANC-1 cells in mice. The results indicated that using a nanoformulation of berberine-loaded lipid nanoparticles enhances the expression of Bax, cleaved caspase-3, and other markers compared to free berberine in pancreatic cancer [72]. Additionally, a recent nanoformulation of 3,3-Diindolylmethane, a plant-derived compound, induced cell death by upregulating Bax and p53 while downregulating Bcl-2 in breast cancer cells [73]. Therefore, the application of nanoformulation delivery system as proposed in our study potentially enhances the anticancer activity via the apoptotic cell death pathway compared to free ELG. Thus, fabrication of the nanoformulation can be a promising selection to use natural therapy as compared to traditional application by direct use of natural compounds.

### 3.12. Inhibition of Receptor Tyrosine Kinases

ELG exhibits a tumour-killing singling pathway by inhibiting receptor tyrosine kinases (RTKs) [74,75]. These findings support the new strategy for cancer prevention and therapy to use small natural agents to target cellular receptors, inhibiting cancer development and progression [76]. Those two receptors play essential roles in cellular signalling in cancers. Figure 10E,F displays the significant inhibition for HER2 and VEGFR2 of HCT-116 and HT-29 cancer cells observed after treatment with MSNTRPELG as compared to ELG and MSN, as well as untreated control. The HER2 maximum inhibitory effect obtained when treated with nanoformulations reached 0.31 ± 0.12-fold change and 0.42 ± 0.13-fold change for HCT-116 and HT-29 cells, respectively. Maximum VEGFR2 inhibition reached 0.37 ± 0.05 and 0.49 ± 0.01-fold change in HCT-116 and HT-29, respectively, compared to seen for ELG and MSN. The results also indicated that either ELG or MSN alone did not show significant effects. It was found that the effectiveness of the tested nanoformulations in inhibiting cellular expression of HER2 and VEGFR2 decreased as follows: MSNTRPELG > free ELG > MSN. Additionally, no significant changes in their expression in BJ-1 normal cells were recorded. This result indicates the negligible response between all forms (nanoformulation, free ELG, and MSN).

### 3.13. Inhibition of B-RAF and K-RAS

To better understand the mechanism of action, we evaluated the ability of the nanoformulations to reduce the protein levels of key molecules involved in cellular signalling pathways in cancer: B-RAF (B-Raf proto-oncogene serine/threonine kinase) and K-RAS (K-Ras proto-oncogene GTPase). B-RAF inhibitors have been effective in cancer therapy, with bevacizumab being the current standard therapy in first-line treatment for BRAF-mutated metastatic colon cancer [46]. B-RAF plays a crucial role in the MAPK/ERK-signalling pathway in cancers, while K-RAS is a member of the RAS gene family and is involved in cell-signalling pathways that regulate cell growth, differentiation, and survival, including the MAPK/ERK and PI3K/AKT pathways.

**Figure 11:**
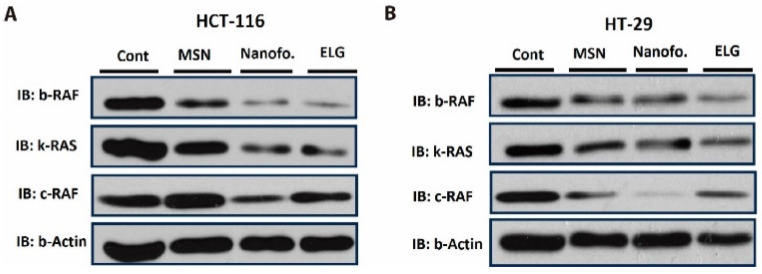
Western blot analysis of B-RAF, K-RAS, and C-RAF protein expression. (**A**) Western blot analysis for HCT-116 and (**B**) Western blot analysis for HT-29 colon cancer cells. The proteins are represented in the upper, middle, and lower panels, respectively. Cells were treated with MSN, nanoformulation, and free ELG at their respective IC50 concentrations and incubated for 48 h. Lane 1: Untreated control; Lane 2: MSN treatment; Lane 3: Nanoformulation treatment; Lane 4: ELG treatment. B-actin was used as loading control (last panel).

The results of Western blot analysis of protein levels in colon cancer cells are shown in Figure 11. In HCT-116 cells (Figure 11A), B-RAF, K-RAS, and C-RAF appeared to be more strongly inhibited by MSNTRPELG compared to free ELG and MSN, relative to untreated control cells. Free ELG also exerted an inhibitory effect, ranking second after the nanoformulation in reducing the expression of these proteins. Inhibition was most pronounced when ELG was combined with MSN in the nanoformulation.

In HT-29 cells (Figure 11B), some variation in effects was observed. The results indicate that MSNTRPELG inhibited C-RAF more strongly than free ELG, MSN, and control cells, whereas free ELG induced a slightly stronger reduction of B-RAF and K-RAS than the nanoformulation and MSN.

**Figure 12:**
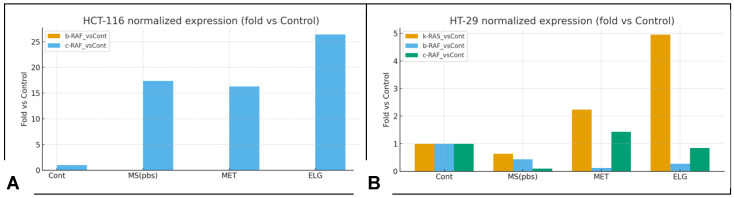
Densitometric quantification of Western blot data is shown in Figure 11. Band intensities of B-RAF, K-RAS, and C-RAF were quantified using ImageJ, normalized to β-actin, and expressed relative to the untreated control (set as 1.0). (**A**) HCT-116 cells; (**B**) HT-29 cells. As only single experiments per condition were available, the densitometric values are presented without inferential statistics.

Densitometric quantification (Figure 12) supported these observations. In HCT-116 cells (Figure 12A), the strongest reduction of B-RAF, K-RAS, and C-RAF was again observed in the MSNTRPELG group, followed by free ELG and MSN. In HT-29 cells (Figure 12B), MSNTRPELG induced the most pronounced inhibition of C-RAF, whereas free ELG produced a comparable or slightly stronger decrease of B-RAF and K-RAS than the nanoformulation. These quantitative profiles corroborate the qualitative observations from Figure 11 and suggest a stronger overall effect of the nanoformulation, particularly on C-RAF. As only single experiments per condition were available, the densitometric results are presented descriptively and should be regarded as tentative, pending confirmation in future studies with biological replicates.

In summary, microscope observations showed a strong attachment of TRP molecules to the MSN surface and confirmed that the functionalization process was successful. The structural studies confirmed successful coating of the nanoparticles with ELG. The MSNTRPELG nanoparticles were well dispersed in deionized water, with a narrow particle size distribution with a mean size of 205.3 ± 15.7 nm. The TRP content was 11 wt.%, the ELG loading content reached about 25 wt.%, and the entrapment efficiency was over 62,5 wt.%. TRP molecules were not only attached to the surface of nanoparticles but also distributed into the mesoporous structure of MSNs.

The MSNTRPELG nanoformulation significantly inhibited HCT-116 and HT-29 colon cancer cells compared to free ELG. The nanoformulation exhibited approximately three-fold stronger anticancer activity than ELG alone, with IC_50_ values of 88.1 ± 0.01 µM for HCT-116 and 77.6 ± 0.1 µM for HT-29, compared to 187.1 ± 0.1 µM and 300.0 ± 0.1 µM for ELG, respectively. Both the nanoformulation and free ELG induced morphological changes from polygonal to rounded shapes, accompanied by cell shrinkage.

The mechanistic insights obtained in this study consistently point to apoptosis as the major pathway of anticancer activity. Quantitative data demonstrated that MSNTRPELG produced significantly stronger inhibition of receptor tyrosine kinases HER2 and VEGFR2 compared to free ELG or MSN while sparing normal BJ-1 cells (Figure 10E,F). These findings are in line with previous reports on the ability of small natural agents to target RTKs and thereby suppress tumour growth and progression [74,75,76].

In addition, densitometric analysis of representative Western blots suggested suppression of B-RAF in both cell lines and of K-RAS in HCT-116 (Figure 11 and Figure 12). Although these data are preliminary due to the lack of biological replicates, they further support the notion that inhibition of RAF/RAS signalling contributes to the induction of programmed cell death.

Taken together, these mechanistic observations indicate that the enhanced anticancer effect of the MSNELG nanoformulation derives primarily from its stronger inhibition of HER2 and VEGFR2, with additional supportive evidence pointing to downregulation of RAF/RAS signalling. Both pathways converge on the promotion of apoptosis, consistent with the observed cell shrinkage and morphological changes, and provide valuable though partly exploratory insights into the therapeutic potential of natural agent–based nanoformulations for colon cancer.

As far as the anticancer efficiency is concerned, the present results are in line with several previous studies that have shown the advantages of nanoformulations containing ELG as a bioactive agent in anticancer therapy. Mady and Shaker [41] encapsulated ELG into poly(ε-caprolactone) (PCL), a biodegradable polymer, showing enhanced anticancer activity against HCT-116 cells when compared to free ELG. This observation may relate to enhanced bioavailability as examined in vivo. Similarly, chitosan-based nanoformulations for ELG were prepared, and they exhibit improved anticancer activity compared to free ELG against breast cancer during in vitro and in vivo studies [43]. Furthermore, El-sonbaty et al. [77] concluded that gallium nanoparticles coated with ELG reduce the viability of MCF-7 human breast cancer cell line compared to ELG alone, as well as produce an effective anticancer effect in vivo rat model.

Our findings suggest some selectivity in the effectiveness of the anticancer effect, with the nanoformulation being more effective in HCT-116 cells, while free ELG showed stronger results in HT-29 cells. In this context, considerable effort is directed towards developing drugs targeting K-RAS mutations in cancer, with limited success until recently, when drugs such as Sotorasib and Adagrasib [78] were developed. The nanoformulation approach represents a promising new therapeutic strategy for treating colorectal cancer through K-RAS inhibition [79]. Furthermore, in our previous study, we demonstrated that a core-shell targeted delivery system based on MSN, carrying colchicine, significantly inhibits B-RAF in HCT-116 colon cancer cells, which is consistent with the current findings in Figure 11A (upper panel) [18]. These results open new possibilities for inhibiting specific targets, such as B-RAF and K-RAS, in cancers like colon cancer, through the development of nanoformulations containing small natural molecules such as ELG.

Further research should include in vivo experiments using animal models of colon cancer. They will yield additional crucial information through studying toxicological, pharmacokinetic, biocompatibility, and biodistribution data, as well as necessary advancements. The following research may also include 3D tumour spheroid evaluation.

## 4. Conclusions

We developed a novel inorganic–organic hybrid nanoformulation to enhance the anticancer efficiency of the natural prodrug ELG. It is composed of mesoporous silica nanoparticles (MSN) functionalized with triptycene (TRP) and loaded with ELG, denoted in the text as MSNTRPELG. The characteristics of the nanoformulation are as follows: average particle size (205.3 ± 15.7 nm), zeta potential (−55.2 ± 1.3 mV), TRP content (11 wt.%), ELG loading content (about 25 wt.%), and entrapment efficiency (62.5 wt.%). It can be supposed that TRP molecules were not only attached to the surface of nanoparticles but also distributed into the mesoporous structure of MSNCOOH. Results obtained for the MSNTRPELG samples indicate the presence of ELG molecules on the surface of nanoparticles, as well as inside the mesoporous structure.

MSNTRPELG significantly inhibited HCT-16 and HT-29 colon cancer lines compared to free ELG. The cytotoxicity was concentration-cell line-dependent. IC50- values were 958.4 ± 0.09 µg/mL for BJ-1, 993.1 ± 0.10 µg/m for HCT-116, and 793.3 ± 0.09 µg/mL for HT-29, respectively. The nanoformulation showed a three times stronger anticancer effect than ELG alone. The IC50 concentration was about 77–88 µM for nanoformulation compared to 300 µM for ELG. Cellular morphology observation showed that nanoformulation and ELG transformed the shapes of the cells from polygonal to circular and resulted in cell shrinkage for HCT-116 and HT-29; however, the effects were stronger for the nanoformulation.

The anticancer mechanism appears to involve apoptosis. The nanoformulation showed a trend toward more efficient inhibition of HER2 and VEGFR2 in HCT-116 and HT-29 cancer cells compared to free ELG. Densitometric analysis of a Western blot suggested a stronger suppression of the B-RAF pathway in both cell lines and of K-RAS in HCT-116, consistent with the induction of programmed cell death. While these findings should be regarded as preliminary, they provide valuable insight into the potential of natural agent-based nanoformulations as promising candidates for colon cancer therapy.

## Figures and Tables

**Table 1:** Sequences of primers for real-time qPCR.

Target Gene	Forward/Reverse
P53	Forward primer 5′-GCTCTGACTGTACCACCATCC-3′ Reverse primer 5′-CTCTCGGAACATCTCGAAGCG-3′
Caspase-3	Forward primer 5′-CTCTCGGAACATCTCGAAGCG-3′ Reverse primer 5′-CAAACTTTTTCAGAGGGGATCG-3′
Bax	Forward primer 5′-AAGCTGAGCGAGTGTCTCCGGCG-3′ Reverse primer 5′-CAGATGCCGGTTCAGGTACTCAGTC-3′
Bcl-2	Forward primer 5′-CTCGTCGCTACCGTCGTGACTTGG-3′ Reverse primer 5′-CAGATGCCGGTTCAGGTACTCAGTC-3′
VEGFR-2	Forward primer 5′-CGTCCGCAAGTGTAAGAA-3′ Reverse primer 5′-AGCAAAAACCCTGTGATT-3′
HER2	Forward primer 5′-AGAGTCACCAGCCTCTGCAT-3′ Reverse primer 5′ -GCAACTCCCAGCTTCACTTT-3′
GAPDH	Forward primer 5′-CGGAGTCAACGGATTTGGTC-3′ Reverse primer 5′-AGCCTTCTCCATGGTCGTGA-3′

**Table 2:** Specific surface area (SSA) and thermogravimetric results of nanoparticles before and after surface functionalization and drug loading.

Nanoformulation	^a^ SSA	^b^ Weight Loss	^c^ Total Drug Loading (TDL)	^d^ Entrapment Efficiency (EE)
MSNCOOH	85.0	20.4	-	-
MSNTRP	30.3	32.1	11.7 (TRP)	-
MSNTRPELG	22.2	57.1	25.0 (ELG)	62.5

^a^ Specific surface area was measured using the BET technique (m^2^/g). ^b^ Weight loss was calculated from the thermogravimetric data, % wt. ^c^ Total drug loading content was calculated using TG data, %. TRP% = % of weight loss of MSNTRP—% of weight loss of MSNCOOH; TDL for ELG into nanoformulation = % of weight loss of MSNTRPELG—% of weight loss of MSNTRP. ^d^ Entrapment Efficiency was calculated using TG data. EE = (% weight loss of MSNTRPELG-% of weight lost of MSNTRP)/% of theoretical drug loading.

## Data Availability

Data for this work are stored in the public repository https://doi.org/10.18150/WAGB4E. URL accessed on 2 October 2025.

## Abbreviations

BJ-1	human fibroblast cell line
B-RAF	B-Raf proto-oncogene serine/threonine kinase
C-RAF	RAF proto-oncogene serine/threonine kinase
DTG	derivative thermogravimetry
DSC	differential scanning calorimetry
EDX	energy dispersive X-ray spectroscopy
ELG	ellagic acid
FE-SEM	field-emission scanning electron microscopy
FTIR-ATR	Fourier transform infrared spectroscopy with attenuated total reflectance
HER2	human epidermal growth factor receptor 2
HCT-116, HT-29	human colon cancer cell lines
IC_50_	half maximal inhibitory concentration
K-RAS	K-Ras proto-oncogene GTPase
MAPK/ERK	mitogen-activated protein kinase/extracellular signal-regulated kinase pathway
MCF-7	human breast cancer cell line
MSN	mesoporous silica nanoparticles
MSNCOOH	carboxyl-functionalized mesoporous silica nanoparticles
MSNTRP	triptycene-functionalized mesoporous silica nanoparticles
MSNTRPELG	triptycene-functionalized mesoporous silica nanoparticles loaded with ellagic acid
MTT assay	3-(4,5-dimethylthiazol-2-yl)-2,5-diphenyltetrazolium bromide assay
NTA	nanoparticle tracking analysis
PEG	poly(ethylene glycol)
PI3K/AKT	phosphoinositide 3-kinase/protein kinase B pathway
PLGA	poly(lactic-co-glycolic acid)
PLGA-PEG	poly(lactic-co-glycolic acid)-block-poly(ethylene glycol) copolymer
RAF/RAS	proto-oncogene signalling pathway
RTKs	receptor tyrosine kinases
STA	simultaneous thermal analysis
STEM	scanning transmission electron microscopy
TEM	transmission electron microscopy
TRP	triptycene
VEGFR2	vascular endothelial growth factor receptor 2
XRD	X-ray diffraction
